# Antagonistic Interactions of Lactic Acid Bacteria from Human Oral Microbiome against *Streptococcus mutans* and *Candida albicans*

**DOI:** 10.3390/microorganisms11061604

**Published:** 2023-06-17

**Authors:** Nikola Atanasov, Yana Evstatieva, Dilyana Nikolova

**Affiliations:** Department of Biotechnology, Faculty of Biology, Sofia University “St. Kliment Ohridski”, 1164 Sofia, Bulgaria; nikolana@uni-sofia.bg (N.A.); y.evstatieva@biofac.uni-sofia.bg (Y.E.)

**Keywords:** lactic acid bacteria, antagonistic activity, *Streptococcus mutans*, *Candida albicans*, biofilm, antioxidant activity

## Abstract

Oral probiotic lactic acid bacteria can exhibit antagonistic activities against pathogens associated with diseases in the oral cavity. Therefore, twelve previously isolated oral strains were assessed for antagonistic evaluation against selected oral test microorganisms *Streptococcus mutans* and *Candida albicans*. Two separate co-culturing analyses were performed, where all tested strains showed the presence of antagonistic activity and four strains, *Limosilactobacillus fermentum* N 2, TC 3-11, and NA 2-2, and *Weissella confusa* NN 1, significantly inhibited *Streptococcus mutans* by 3–5 logs. The strains showed antagonistic activity against *Candida albicans*, and all exhibited pathogen inhibition by up to 2 logs. Co-aggregation capability was assessed, showing co-aggregative properties with the selected pathogens. Biofilm formation and antibiofilm activity of the tested strains against the oral pathogens were assayed, where the strains showed specificity in self-biofilm formation and well-expressed antibiofilm properties by most of them above 79% and 50% against *Streptococcus mutans* and *Candida albicans*, respectively. The tested LAB strains were assayed by a KMnO_4_ antioxidant bioassay, where most of the native cell-free supernatants exhibited total antioxidant capacity. These results show that five tested strains are promising candidates to be included in new functional probiotic products for oral healthcare.

## 1. Introduction

The human oral cavity is the second most microbiome diverse after the gastrointestinal tract [1,2]. The balance between coexisting microbial species is essential for oral health but is often difficult to maintain. When the equilibrium is disrupted, mainly streptococcal and yeast pathogens start predominating and supplanting their oral microbiota, and different oral diseases can occur [3].

*Streptococcus mutans* is a facultative anaerobic, gram-positive coccus commonly found in the human oral cavity and is the core microorganism contributor in the dental caries etiology [4,5]. Dental caries is the most widespread biofilm-mediated disease, which occurs when environmental changes in the oral cavity favour the growth of cariogenic bacteria. *S. mutans* can efficiently convert a wide range of carbohydrates to organic acids, demineralizing agents for tooth enamel. Also, its ability to tolerate environmental stresses and produce extracellular polysaccharides and glycogen stores promotes bacterial adhesion and accumulation with other microorganisms on the dental surface [6].

*Candida albicans* is an opportunistic yeast pathogen normally detected in the human gastrointestinal tract [7]. Generally commensal, it can become pathogenic under various compromised conditions of the immune system and is one of the few species of the *Candida* genus that contributes to oral and vaginal candidiasis infections [8]. Moreover, its pathogenicity is caused by disrupting the equilibrium of the conventional microbiota and destroying the epithelial protective barrier. In the course of infection, the formation and accumulation of biofilm greatly influence pathogenicity. As a dimorphic fungus, the ability to morphologically transform from yeast to filamentous cell growth is the core event in the disease process [9].

Lactic acid bacteria (LAB) are a large group used as probiotics that manifest health benefits to humans and are generally recognized as safe (GRAS). They can possess characteristics such as immunomodulation, protection against harmful microorganisms and improving the balance of the host microbiota [10,11]. To exhibit their effectiveness, oral probiotics should form aggregates on oral tissues to competitively inhibit pathogen growth [12,13] and/or slow down the colonization of potentially pathogenic species [14]. It is suggested that probiotic microorganisms have a vital participation in the prevention of different oral diseases when consumed regularly [15]. Intensive research on the properties of probiotics in maintaining ecological equilibrium and efficiently normalizing the oral microbiota has been an essential subject for many years [16,17,18,19,20].

The direct antimicrobial interaction of probiotics is one of the important properties against undesirable microbiota in the oral cavity. The competitive exclusion of pathogens due to the physical presence of LAB and enhancing the barrier function of the mucosal tissue are essential aspects of maintaining homeostasis [21]. The production of antimicrobial metabolites, including organic acids, exopolysaccharides, bacteriocins and anti-adherence biosurfactants, possess antimicrobial properties [22,23,24]. Therefore, studying the direct antimicrobial interactions of probiotic LAB with oral pathogenic microorganisms is essential.

Co-aggregation is an essential mechanism in biofilm formation and a significant factor in the development of dental plaque [25,26]. The co-aggregative properties of LAB can activate the formation of a barrier that inhibits the colonization of pathogenic microorganisms [27]. The co-aggregative abilities of LAB with orally associated pathogens are one of the main characteristics of their antagonistic activity in the oral cavity.

Naturally, biofilms are a form of cell self-immobilization resulting from microbial attachment to solid biotic/abiotic surfaces in a submerged environment. LAB can form high cell-density biofilms, which allows them to withstand stressful conditions, including changes in pH or nutrient limitation [28]. Most importantly, LAB biofilms have a significant role in exerting antagonistic functions against various pathogenic microorganisms. By creating a barrier for pathogens in either planktonic or biofilm states, LAB can inhibit their adherence to the mucosal tissue [29]. Pathogenic biofilms, on the other hand, are one of the main causes of diseases and increased antibiotic resistance. Within the biofilm, pathogens develop resistance to different environmental conditions and substances, including temperature, antimicrobial compounds, host oxygen radicals and phagocytes, and proteases [30]. Also, pathogen cells in biofilms possess self-protective capabilities, low metabolic activity and are less subjected to mutations [31]. In this matter, the capacity of LAB to produce biofilms is essential, as well as their ability to inhibit the formation of pathogenic biofilms.

The Food and Agriculture Organization of the United Nations (FAO), the World Health Organization (WHO) and The International Scientific Association for Probiotics and Prebiotics (ISAPP) defined probiotics as “live microorganisms that, when administered in adequate amounts, confer a health benefit on the host” [32,33]. Probiotic strains may have antioxidant characteristics through chelating metal ions, production of metabolites, downregulation of enzymes from reactive oxygen species (ROS), and upregulation of antioxidant activities [34]. These mechanisms can improve the host’s defence against oxidative stress and promote the prevention of different diseases [35]. In most living organisms, ROS are eliminated by enzymatic and non-enzymatic defence and repair systems that maintain homeostasis against oxidative stress [36]. But if an imbalance in ROS production and antioxidant ability is present, damage to cells and tissues may occur. LAB can produce antioxidant enzymes, such as superoxide dismutase (SOD) and can stimulate the antioxidant system of the host and enhance the activities of antioxidases [37], which makes their antioxidant properties important to be studied.

The addition of probiotics in different products is one of the strategies of pharmaceutical and food industries to improve consumer health, and extensive in vivo studies focus on LAB to treat various oral conditions [3]. Data on clinical studies show that administering probiotic products can improve chronic periodontitis [38], reduce periodontal pocket depth [39], reduce gingivitis and plaque indices [40], and treat various dysbiosis-inducing pathological conditions, including dental caries, periodontitis and halitosis. Probiotics can be included in different vehicles, such as chewing gum, tablets, capsules, oil drops, milk, sachet, and lozenges, which, when applied, can have positive effects on oral health [3].

The selected group of LAB strains used in the present study was evaluated for probiotic properties in a previous study. According to the obtained data, these strains show probiotic potential regarding survival and growth in the oral cavity environment and the transition to the next parts of the GIT. An expressed antimicrobial activity against common Gram+ and Gram-pathogens, including *Escherichia coli*, *Bacillus subtilis* and *Bacillus cereus*, was reported. Also, the evaluated strains exhibited an expressed ability to bind to mucin, measured at 5 logs CFU/mL [41].

In this context, this work aimed to assess the properties of previously isolated and selected groups of oral LAB strains [41] regarding their direct antagonistic activity against pathogenic microorganisms, *S. mutans* and *C. albicans*, associated with diseases in the oral cavity by co-culturing methods. To evaluate their ability to co-aggregate with them, their capacity to self-form biofilms, their ability to inhibit biofilm formation of the selected pathogens, and their antioxidant capacity.

## 2. Materials and Methods

### 2.1. Microorganism Strains and Cell-Free Supernatant Preparation

The studied LAB strains were previously isolated from human oral cavity microbiome and identified as *Limosilactobacillus fermentum* N 2, *Limosilactobacillus fermentum* N 4-5, *Weisella confusa* AG 2-6, *Latilactobacillus curvatus* KG 12-1, *Limosilactobacillus fermentum* TC 3-11, *Lactobacillus delbrueckii* subsp. *sunkii* VG 1, *Lactobacillus delbrueckii* subsp. *lactis* VG 2, *Lactobacillus delbrueckii* subsp. *lactis MK* 13-1, *Weisella confusa* NN 1, *Lacticaseibacillus rhamnosus* NA 1-8, *Limosilactobacillus fermentum* NA 2-2 and *Lacticaseibacillus paracasei* AV 2-1 [41]. The test microorganisms *Streptococcus mutans* ATCC 25175 and *Candida albicans* ATCC 10231 were selected as test pathogens for antagonistic activity evaluation. *S. mutans* was cultivated in Brain Heart infusion (BHI) broth/agar (Sigma-Aldrich, St. Louis, MO, USA) and Tryptone Yeast Extract Cystine w/Sucrose, supplemented with Bacitracin (TYCSB) agar (HiMedia, Mumbai, India). *C. albicans* was cultivated in Malt Extract (ME) broth/agar (Oxoid, Basingstoke, UK).

The tested LAB strains were cultured overnight at 37 °C in MRS broth, and cell-free supernatants (CFSs) were collected by centrifugation at 6000× *g* for 10 min at 4 °C. One CFS batch was stored as native, and a second batch was neutralized to pH 7 with 1 N NaOH.

### 2.2. Antagonistic Activity Assay by Co-Cultivation

The antagonistic activity was assayed by two different methods. The agar spot assay was used firsthand [21]. Overnight cultures of the tested LAB were brought to 10^9^ CFU/mL, spotted on MRS agar plates, and cultivated anaerobically at 37 °C for 24 h to develop spots. Cultures of the test pathogens were standardized to 10^8^ CFU/mL and mixed in 0.7% agar media at 45–50 °C and poured gently over the spotted agar plates. The petri dishes were cultivated anaerobically at 37 °C for *S. mutans* and aerobically at 30 °C for *C. albicans* for 24 h, and the presence of inhibition zones was reported.

A co-culturing assay was performed based on Denkova et al. [42] with some modifications. The tested LAB strains were incubated overnight, centrifuged at 6000× *g* for 10 min and resuspended in the respective media for the test pathogens. The test pathogens were standardized to 10^8^ CFU/mL, and 0.5 mL of LAB culture was mixed with 0.5 mL of test pathogen and inoculated in 9 mL of the respective medium. 0.5 mL of the test pathogen inoculated in a 9.5 mL medium was used as a control. The co-cultivation was performed at 37 °C for *S. mutans* and at 30 °C for *C. albicans* for 48 h. Samples were taken at 24 and 48 h, diluted by ten-fold serial dilutions, and then cultivated in the respective agar media anaerobically at 37 °C for *S. mutans* and aerobically at 30 °C for *C. albicans* for 48 h. The results were reported as CFU/mL from the cell count of the test pathogens.

### 2.3. Co-Aggregation

The LAB strains were assayed for co-aggregation capability [43] with the oral test pathogens *S. mutans* and *C. albicans*. The tested LAB cultures were centrifuged at 6000× *g* for 10 min and washed twice with PBS. LAB and test-pathogen cultures were brought to 10^8^ CFU/mL, and equal volumes (1:1, *v*/*v*) of different LAB and pathogens were mixed. Aliquots were measured with SPECTROstar^®^ Nano Microplate Reader (BMG LABTECH, Ortenberg, Germany) at 600 nm (A_initial_). The cell mixtures were then incubated at 37 °C for *S. mutans* and 30 °C for *C. albicans* statically for 4 h, and uppermost fractions were measured (A_final_). Cultures of both test pathogens alone were used as autoaggregation controls. The co-aggregation percentage was calculated with the equation:

Co-aggregation (%) = (A_initial_ − A_final_)/(A_initial_) × 100 (1)

### 2.4. Biofilm Formation

The crystal violet method was used to evaluate the biofilm-producing potential of the tested LAB strains [44]. Aliquots of 100 µL overnight LAB cultures were added to the wells of a 96-well microplate, previously coated with MRS broth, and the plate was incubated at 37 °C for 24 h. After incubation, the non-attached cells were removed, and the wells were washed three times with a phosphate-buffered solution (PBS). The cell biofilms were stained with 100 µL 0.1% crystal violet for 30 min, then washed five times with PBS to remove the excess stain. The plate was then left to dry out for 30 min, and the absorbance was measured at 640 nm using the SPECTROstar^®^ Nano Microplate Reader. Wells inoculated with MRS medium only were used as a negative control. The results were presented as percent by subtracting the absorbance of the negative control from the absorbance of each inoculated well.

### 2.5. Antibiofilm Activity

The tested LAB strains were evaluated by anti-biofilm activity assay [44] against the test pathogens *S. mutans* and *C. albicans*. Aliquots of 100 µL overnight cultures of the pathogens were added to the wells of 96-well microtiter plates and 100 µL of neutralized CFS (pH 7) of the tested LAB strains. The plates were incubated at 37 °C for *S. mutans* and 30 °C for *C. albicans* for 24 h, then the medium with non-attached cells was discarded, and the wells were washed twice with PBS. The formed biofilms were fixed with 200 µL methanol for 10 min and stained with 200 µL 0.1% crystal violet for 10 min. After washing three times with distilled water, the crystal violet attached to the biofilms was dissolved with 200 µL 33% acetic acid. The absorbance was measured at 590 nm using SPECTROstar^®^ Nano Microplate Reader. Positive control comprised test-pathogen cultures without CFS addition, BHI medium, and Malt extract medium only as a negative control. The percentage of biofilm inhibition was calculated by the following equation:

Biofilm inhibition (%) = (A_growth control_ − A_sample_)/(A_growth control_) × 100 (2)

### 2.6. Antioxidant Capacity

The total antioxidant capacity of the tested LAB strains was assayed by the potassium permanganate (KMnO_4_) agar method [45]. 2% agar was dissolved in hot sterile distilled water and supplemented with 0.003 M KMnO_4_ to a 0.5 mmol L^−1^ concentration. The KMnO_4_ agar was poured into petri dishes, and wells were made with a cork borer after solidification. The wells were then inoculated with 80 µL CFS, and the agar plates were stored at 4 °C in the dark. Measurements were taken at 10 min, 30 min, 1 h, 4 h, and 24 h to develop discoloured zones. Wells inoculated with MRS medium only were used as a control. The antioxidant activity was measured by subtracting the discoloured zone diameter of the control from the zone diameter of each sample.

### 2.7. Data Analysis

All experiments were performed in triplicate. The obtained data were analyzed by Microsoft Excel built-in functions, and the results were expressed as mean ± standard deviation. Two-tailed Student’s *t*-test was carried out as a statistical evaluation to detect differences between controls and samples. *p*-value of *p* < 0.05 was considered as statistically significant difference. One-way ANOVA analysis was utilized for statistical evaluation of the co-aggregation, biofilm formation, antibiofilm activity and antioxidant capacity. *p*-value of *p* < 0.01 was considered a statistically significant difference. Pearson Correlation analysis was carried out as a statistical comparison of the relationship between the co-cultivation, co-aggregation and antibiofilm activity analyses.

## 3. Results

### 3.1. Antagonistic Activity Assay by Co-Cultivation

The direct interactions need to be studied for LAB to exclude pathogens in the environment of the oral cavity. From the spot assay, inhibition zones were reported from all of the tested LAB strains against *S. mutans*. Against *C. albicans*, inhibition zones were observed from eight of the studied strains: *L. fermentum* N2, N 4-5, TC 3-11 and NA 2-2, *L. delbrueckii* subsp. *lactis* VG 2, *W. confusa* NN 1, *L. rhamnosus* 1-8, and *L. paracasei* AV 2-1 (Table 1).

**Figure 1:**
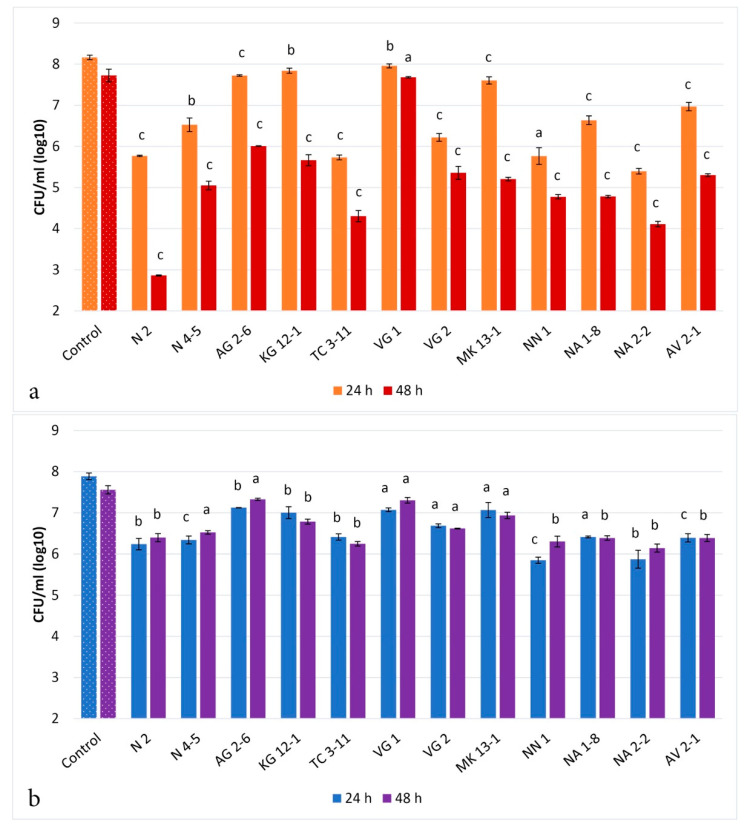
Antagonistic activity of the tested LAB strains in direct co-cultivation with *S. mutans* (**a**) and *C. albicans* (**b**). Values are expressed as mean ± standard deviation. Statistical analysis was performed by Student’s *t*-test: a—nonsignificant (*p* > 0.05); b and c—significant (*p* < 0.05 and *p* < 0.01, respectively). Strains: *Limosilactobacillus fermentum* N 2, *Limosilactobacillus fermentum* N 4-5, *Weisella confusa* AG 2-6, *Latilactobacillus curvatus* KG 12-1, *Limosilactobacillus fermentum* TC 3-11, *Lactobacillus delbrueckii* subsp. *sunkii* VG 1, *Lactobacillus delbrueckii* subsp. *lactis* VG 2, *Lactobacillus delbrueckii* subsp. *lactis MK* 13-1, *Weisella confusa* NN 1, *Lacticaseibacillus rhamnosus* NA 1-8, *Limosilactobacillus fermentum* NA 2-2 and *Lacticaseibacillus paracasei* AV 2-1.

The results are a primary screening that the tested strains exhibit antagonistic properties against the selected oral pathogens. The direct co-culturing assay was performed to quantitatively evaluate the antagonistic activity of the tested LAB strains against the selected pathogens (Figure 1).

The results from the co-culturing assay showed that at 24 h *L. fermentum* N2, N 4-5, TC 3-11 and NA 2-2, *L. delbrueckii* subsp. *lactis* VG 2, *W. confusa* NN 1, *L. rhamnosus* NA 1-8, and *L. paracasei* AV 2-1 exhibit antagonistic activity against *S. mutans* by decreasing the viability of the pathogen by 1–3 logs. At 48 h antagonistic activity was reported from more of the tested LAB strains—*L. fermentum* N2, N 4-5, TC 3-11 and NA 2-2, *L. delbrueckii* subsp. *lactis* VG 2 and MK 13-1, *W. confusa* AG 2-6 and NN 1, *L. rhamnosus* 1-8, and *L. paracasei* AV 2-1 exhibited a decrease in pathogen live cell number by 2–5 logs. Only one strain *L. delbrueckii* subsp. *sunkii* VG 1 had no activity against *S. mutans* (Figure 1a). All of the tested LAB strains showed slightly expressed antagonistic activity against *C. albicans* by decreasing the viability of the pathogen by 1–2 logs until 48 h (Figure 1b).

### 3.2. Co-Aggregation

**Figure 2:**
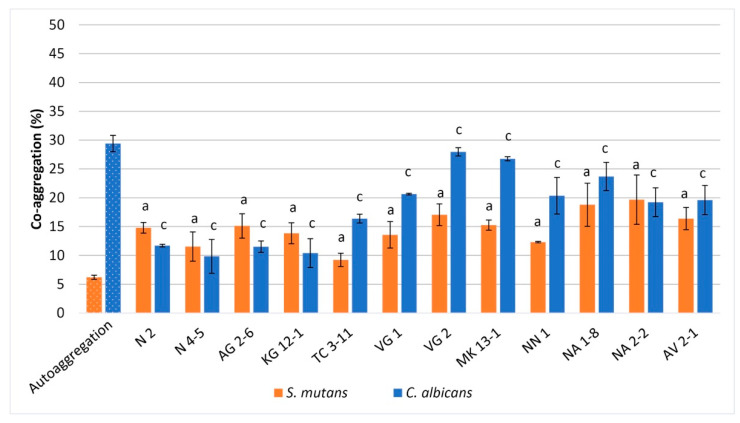
Co-aggregation of the tested LAB strains with *S. mutans* and *C. albicans*. Values are expressed as mean ± standard deviation. Statistical analysis was performed by One-way ANOVA: a—nonsignificant (*p* > 0.05); c—significant (*p* < 0.01). Strains: *Limosilactobacillus fermentum* N 2, *Limosilactobacillus fermentum* N 4-5, *Weisella confusa* AG 2-6, *Latilactobacillus curvatus* KG 12-1, *Limosilactobacillus fermentum* TC 3-11, *Lactobacillus delbrueckii* subsp. *sunkii* VG 1, *Lactobacillus delbrueckii* subsp. *lactis* VG 2, *Lactobacillus delbrueckii* subsp. *lactis MK* 13-1, *Weisella confusa* NN 1, *Lacticaseibacillus rhamnosus* NA 1-8, *Limosilactobacillus fermentum* NA 2-2 and *Lacticaseibacillus paracasei* AV 2-1.

Co-aggregation represents the intercellular adhesion properties of different microorganisms [46]. All subjected to co-aggregation LAB strains indicated co-aggregative properties with the selected oral test pathogens. With *S. mutans*, all tested LAB strains exhibited co-aggregation in the range of 9.22–19.67% higher than the autoaggregation of the test pathogen, measured at 6.21%. The highest co-aggregation was observed from *L. fermentum* NA 2-2 and *L. rhamnosus* NA 1-8, measured at 19.67% and 18.78%, respectively. The lowest co-aggregation was observed from *L. fermentum* TC 3-11, measured at 9.22% (Figure 2).

With *C. albicans*, all tested LAB strains exhibited co-aggregation in the range of 9.83–27.97%, lower than the autoaggregation of the test pathogen, measured at 29.41%. *L. delbrueckii* subsp. *lactis* VG 2 and MK 13-1 showed the highest co-aggregation with the test pathogen at 27.97% and 26.76%, respectively. The lowest co-aggregation was shown from *L. fermentum* N 2 and N 4-5, *W. confusa* AG 2-6, and *L. curvatus* KG 12-1, measured between 9.83% and 11.70% (Figure 2).

### 3.3. Biofilm Formation

**Figure 3:**
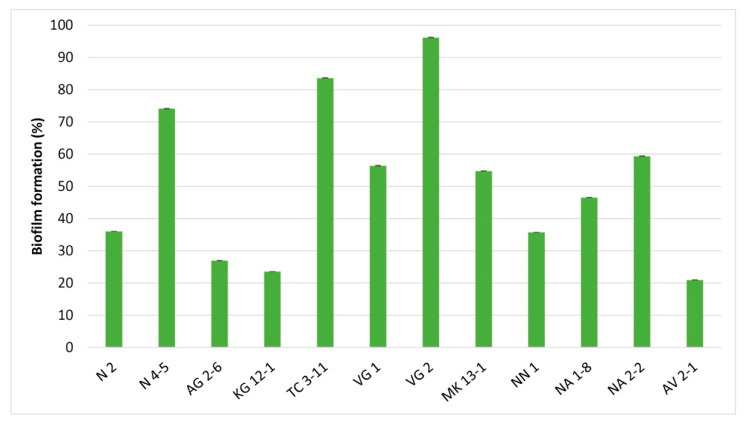
Percent of biofilm formation by the tested LAB strains. Values are expressed as mean ± standard deviation. Statistical analysis was performed by One-way ANOVA (*p* < 0.01). Strains: *Limosilactobacillus fermentum* N 2, *Limosilactobacillus fermentum* N 4-5, *Weisella confusa* AG 2-6, *Latilactobacillus curvatus* KG 12-1, *Limosilactobacillus fermentum* TC 3-11, *Lactobacillus delbrueckii* subsp. *sunkii* VG 1, *Lactobacillus delbrueckii* subsp. *lactis* VG 2, *Lactobacillus delbrueckii* subsp. *lactis MK* 13-1, *Weisella confusa* NN 1, *Lacticaseibacillus rhamnosus* NA 1-8, *Limosilactobacillus fermentum* NA 2-2 and *Lacticaseibacillus paracasei* AV 2-1.

The crystal violet staining assay can be used as an indirect method for determining the amount of accumulated bacterial biofilm [47]. All tested LAB strains showed the ability to form biofilms with differentiating percent (Figure 3). *L. delbrueckii* subsp. *lactis* VG 2 expressed a percentage of biofilm formation above 96%, the highest among the tested LAB strains in this in vitro analysis. For *L. fermentum* TC 3-11 and N 4-5 strains, well-expressed formation of biofilm was also reported, measured above 74%. *L. fermentum* NA 2-2, *L. delbrueckii* subsp. *sunkii* VG 1 and *L. delbrueckii* subsp. *lactis* MK 13-1 were the strains that exhibited biofilm formation ability, measured above 50%.

### 3.4. Antibiofilm Activity

**Figure 4:**
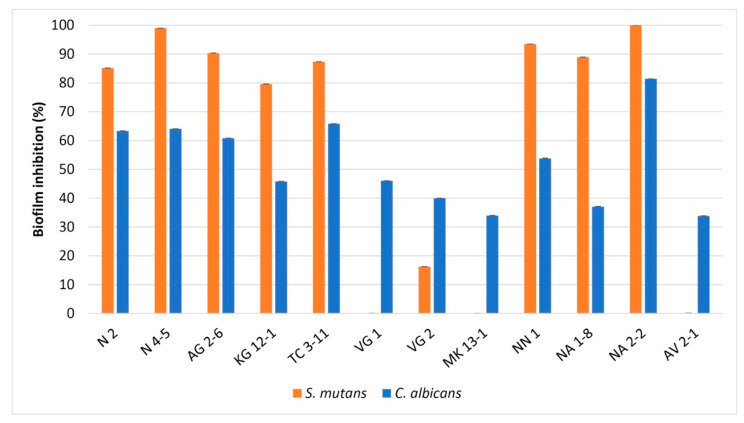
Percent of biofilm inhibition by the tested LAB strains against *S. mutans* and *C. albicans*. Values are expressed as mean ± standard deviation. Statistical analysis was performed by One-way ANOVA (*p* < 0.01). Strains: *Limosilactobacillus fermentum* N 2, *Limosilactobacillus fermentum* N 4-5, *Weisella confusa* AG 2-6, *Latilactobacillus curvatus* KG 12-1, *Limosilactobacillus fermentum* TC 3-11, *Lactobacillus delbrueckii* subsp. *sunkii* VG 1, *Lactobacillus delbrueckii* subsp. *lactis* VG 2, *Lactobacillus delbrueckii* subsp. *lactis MK* 13-1, *Weisella confusa* NN 1, *Lacticaseibacillus rhamnosus* NA 1-8, *Limosilactobacillus fermentum* NA 2-2 and *Lacticaseibacillus paracasei* AV 2-1.

The ability of oral LAB to inhibit biofilms of pathogens in the oral cavity is essential for their application as oral probiotics. From the held antibiofilm assay, nine of the tested LAB strains exhibited inhibition of biofilm formation by *S. mutans*, and all strains inhibited biofilm formation of *C. albicans* (Figure 4). Against *S. mutans*, eight of the tested strains exhibited definite biofilm inhibitory activity, and only three did not exhibit inhibition in the conditions of the in vitro analysis. The strain *L. fermentum* NA 2-2 showed excellent pathogen biofilm inhibition properties by 100%. Following this result, *L. fermentum* N 2, N 4-5 and TC 3-11, *W. confusa* AG 2-6 and NN 1, *L. curvatus* KG 12-1, and *L. rhamnosus* NA 1-8 also expressed high biofilm inhibition properties, measured above 79%. From the tested LAB strains, *L. delbrueckii* subsp. *lactis* VG 2 showed to possess the lowest biofilm inhibition. For *L. delbrueckii* subsp. *sunkii* VG 1, *L. delbrueckii* subsp. *lactis* MK 13-1 and *L. paracasei* AV 2-1, no biofilm inhibition properties were observed in this in vitro analysis.

Against *C. albicans*, all of the tested LAB strains possess antibiofilm activity. The strain *L. fermentum* NA 2-2 exhibited the highest biofilm inhibition, above 81%. This strain, along with *L. fermentum* N 2, N 4-5 and TC 3-11, and *W. confusa* AG 2-6, showed to inhibit biofilm formation that exceeds 60% (Figure 4).

### 3.5. Antioxidant Capacity

**Figure 5:**
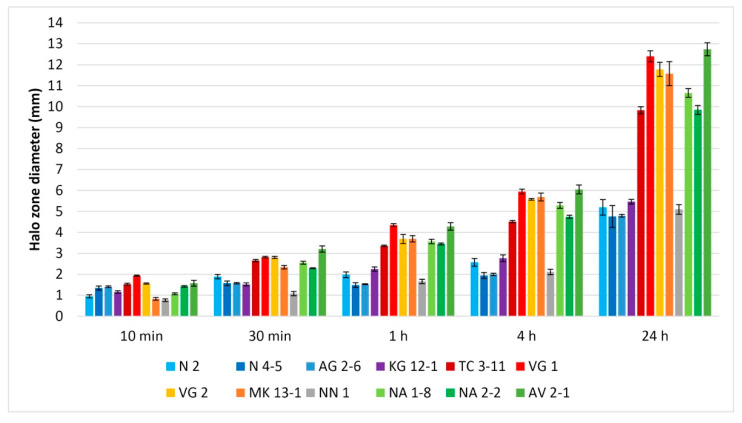
Total antioxidant capacity of the tested LAB strains. Values are expressed as mean ± standard deviation. Strains: *Limosilactobacillus fermentum* N 2, *Limosilactobacillus fermentum* N 4-5, *Weisella confusa* AG 2-6, *Latilactobacillus curvatus* KG 12-1, *Limosilactobacillus fermentum* TC 3-11, *Lactobacillus delbrueckii* subsp. *sunkii* VG 1, *Lactobacillus delbrueckii* subsp. *lactis* VG 2, *Lactobacillus delbrueckii* subsp. *lactis MK* 13-1, *Weisella confusa* NN 1, *Lacticaseibacillus rhamnosus* NA 1-8, *Limosilactobacillus fermentum* NA 2-2 and *Lacticaseibacillus paracasei* AV 2-1.

The antioxidant activity of LAB is essential to be studied as their antioxidant enzyme production can protect the host from damage from free radicals that have a role in the development of many chronic diseases [48]. The used method is recently adapted for LAB and estimates the total antioxidant capacity of their native CFS. The assessment of the antioxidant capacity of the tested LAB strains was performed in addition to their previously evaluated probiotic properties [41]. All tested LAB CFSs exhibited antioxidant capacity throughout the experiment (Figure 5). The organic antioxidant compounds react with the KMnO_4,_ and distinct transparent zones can be observed. At 10 min, small occurring halo zones can already be seen. At 30 min, 1 h and 4 h, the zones have clear boundaries and the CFSs from *L. fermentum* TC 3-11 (pH 3.72) and NA 2-2 (pH 3.92), *L. delbrueckii* subsp. *sunkii* VG 1 (pH 3.42), *L. delbrueckii* subsp. *lactis* VG 2 (pH 3.41) and MK 13-1 (pH 3.54), *L. rhamnosus* NA 1-8 (pH 3.60), and *L. paracasei* AV 2-1 (pH 3.44) exhibited well-expressed antioxidant capacity. At 24 h the discoloured zones appear with more diffused boundaries. Two of the tested strains *L. delbrueckii* subsp. *sunkii* VG 1 and *L. paracasei* AV 2-1 showed the highest antioxidant capacity among the tested strains. Followed by *L. fermentum* TC 3-11 and NA 2-2, *L. delbrueckii* subsp. *lactis* VG 2 and MK 13-1, and *L. rhamnosus* NA 1-8. These strains also showed high antioxidant capacity. The CFSs from *L. fermentum* N 2 (pH 3.99) and N 4-5 (pH 4.00), *W. confusa* AG 2-6 (pH 3.89) and NN 1 (pH 3.92), and *L. curvatus* KG 12-1 (pH 3.90) exhibited lower antioxidative properties throughout the experiment (Figure 5). It can be noted that between 30 min and 4 h an even increase in the halo zone diameter is observed.

## 4. Discussion

Both *S. mutans* and *C. albicans* significantly contribute to the pathogenesis of caries, candidiasis, and periodontitis infections [5,49]. Lactobacilli also play a crucial role in the oral ecosystem by contributing to oral health [50]. The obtained data from the co-culturing assay shows significant inhibition of *S. mutans* from most of the tested LAB strains (Figure 1a). Most importantly, *L. fermentum* N 2, TC 3-11 and NA 2-2, and *W. confusa* NN 1 exhibited the highest impact on the cell density of the pathogen throughout the co-cultivation. For *C. albicans*, it can be observed that the same eight strains that showed antagonistic activity in the spot analysis exhibited better activity in the co-cultivation assay (Figure 1b).

On the other hand, the yeast pathogen proves its opportunistic status [51] as only two of the LAB strains, *L. fermentum* NA 2-2 and *W. confusa* NN 1, showed up to 2 logs of inhibition at 24 h. The agar spot assay has already been described as a modified agar spot-on-lawn assay and reported by several authors as a simple method for determining the presence of antagonistic activity of probiotic bacteria [21]. The co-culturing assay is used as an in vitro method for reporting the direct antagonistic activity of potential probiotic bacteria by enumerating the cell density of pathogenic microorganisms, specifically oral pathogens such as *S. mutans* and *C. albicans*. In the assay performed by Denkova et al. [42], the authors used the *Lactobacillus acidophilus* strain against *E. coli*, *Staphylococcus* and *Salmonella* sp., where the LAB strain reduced pathogenic cell density by 1–2 logs until 24 h and 4–8 logs until 48 h of the co-culturing. In a study by Chen et al. [52], *L. fermentum* and *Ligilactobacillus salivarius* showed definite inhibition of oral cariogenic and periodontal bacteria, including *S. mutans*, *Streptococcus sanguinis* and *Porphyromonas gingivalis*. In another study, Mann et al. [53] used *Lactobacillus gasseri*, which reduced the cell density of oral streptococci, *Porphyromonas* sp. and *Fusobacterium nucleatum* as low as 10^5^ CFU/mL. In a previous study by Denkova et al. [54], the authors evaluated the antagonistic activity of *L. acidophilus* and *Lactobacillus delbrueckii* subsp. *bulgaricus* strains reduced the cell density of *C. albicans* by 2 logs at 24 h and 2 to 3 logs at 48 h of co-culturing. A study by Vazquez-Munoz et al. [55] assessed a *Lactobacillus johnsonii* strain that showed inhibition of *C. albicans* by 10.1% in a 1:1 cell ratio co-cultivation for 24 h. It is important to note that eleven of the LAB strains included in our study possess expressed antagonistic activity against *S. mutans*, which has a statistically significant decrease in the cell density of the pathogen. The obtained data from the co-cultivation assay on the tested LAB strains in our study support and substantially add to the results in the studies of other authors cited above. The scientific publications are limited to research on LAB being evaluated in co-cultivation assays with cariogenic and periodontal pathogens. Research on *C. albicans*, however, is extensive due to the opportunistic nature of the pathogen and its widespread human infection niches [51]. As a novelty, our research evaluated *L. curvatus*, *L. rhamnosus*, *L. paracasei*, and *W. confusa* strains, previously not studied in co-culturing techniques with oral pathogenic microorganisms. Our research provides confirmation of the direct interactions of the LAB included in our study with oral pathogenic microorganisms, especially with *S. mutans*, regarding their antagonistic properties in the composition of the oral microbiota.

The determination of the potential aggregative properties of LAB is influenced by internal and environmental factors [56]. Co-aggregation has been observed between microbial species in the oral microbiome’s composition. It is suggested that LAB, which can co-aggregate with oral pathogens, may exert an important host defence mechanism against infection development [57]. From the obtained data, all tested LAB strains in our study possess the property to co-aggregate with *S. mutans*, and most of them showed well-expressed co-aggregation with *C. albicans* (Figure 2). Statistically, no significant difference was reported for the tested LAB strains in co-aggregation with *S. mutans*. However, statistically significant differences among the tested strains were reported in co-aggregation with *C. albicans*. This shows specificity in their co-aggregative properties with the yeast pathogen and can be treated as a strain-specific property. It can be observed that three of the tested LAB strains possess well-expressed co-aggregation with *C. albicans*: *L. delbrueckii* subsp. *lactis* VG 2 and MK 13-1, and *L. rhamnosus* NA 1-8. Strain specificity was reported by other authors as well. LAB strains studied by Ciandrini et al. [58], including *L. paracasei* and *L. rhamnosus*, showed co-aggregative properties with *S. mutans* in the range of 6.32–20.93%. In a study by Lai et al. [11], five LAB strains were evaluated, and co-aggregation with *S. mutans* was reported between 15.93 and 62.25%. In a study by Aarti et al. [59], the evaluated *Lactiplantibacillus pentosus* strain exhibited 37.1% co-aggregative properties with *C. albicans*. Malfa et al. [60] used a multistrain formulation of LAB strains, *L. rhamnosus* strain included, and observed high co-aggregation capability with *C. albicans*.

The results from the biofilm formation assay show significant differences among strains of the same species (Figure 3). Biofilm formation among the four *L. fermentum* strains ranges between 36% and 83%. From the tested *L. delbrueckii* strains, while VG 1 and MK 13-1 exhibited similar biofilm formation, for VG 2, the formed biofilm was noticeably higher. The two *W. confusa* strains AG 2-6 and NN 1 also showed different percentages of biofilm formation. This data suggests biofilm formation could be a strain-specific property among LAB representatives. This could also be influenced by environmental factors and microbial composition in the oral cavity. In a study by Jha et al. [44], the biofilm formation among the six tested LAB strains varied between 19.32 and 85.84%. Gómez et al. [61] described similar strain dependency between the studied *Lactococcus lactis* and *L. curvatus* strains. Kubota et al. [29] evaluated 46 LAB strains, including eight *Lactiplantibacillus plantarum* and four *Levilactobacillus brevis* strains, showing differences in biofilm formation capability among the same species. The formation of biofilms is an important mechanism by which LAB manifest their beneficial properties by adhering and accumulating to mucosal tissue. This trait also increases the antagonistic effects against pathogenic microorganisms in the gastrointestinal tract [62].

*S. mutans* produces cell-wall anchored proteins that facilitate binding to *C. albicans*, which successively assist streptococcal colonization and further caries development from the formed biofilm [5,63,64]. In the antibiofilm assay, *L. fermentum* NA 2-2 exhibited the highest biofilm inhibition against both pathogens, possessing the capacity to prevent the accumulation of pathogenic biofilms in vitro (Figure 4). From the results, it can be suggested that antibiofilm activity against the selected oral pathogens could be a species-related activity. All four *L. fermentum* strains’ biofilm inhibition was high against both pathogens. *W. confusa* AG 2-6 and NN 1 showed similar percentages of inhibition against the test pathogens. The similarity in biofilm inhibition against *C. albicans* was also shown by the tested *L. delbrueckii* subsp. *sunkii* VG 1, and *L. delbrueckii* subsp. *lactis* VG 2 and MK 13-1 strains. In a study by Patel et al. [65], the authors evaluated glycolipid biosurfactant derived from a newly isolated *L. rhamnosus* strain which was effective in inhibiting biofilms of *E. coli*, *B. subtilis*, *Pseudomonas aeruginosa* and *Staphylococcus aureus*. In the study by Jha et al. [44], the six tested LAB strains showed biofilm inhibition properties against *S. mutans* in the 2.09–15.07% range. Wasfi et al. [66] studied four *Lactobacillus* sp. strains, which showed antibiofilm activity against *S. mutans* from 24.7 to 47%. The constant presence of LAB strains can potentially increase their intervention with pathogens. In that matter, LAB species normally found in the composition of the oral microbiome possess higher antibiofilm activity, as seen from the results of our study. Wu et al. [67] evaluated sixty-four strains of *L. salivarius* from human saliva, inhibiting biofilm formation of *S. mutans* up to 69%. Krzyściak et al. [68] showed that *L. salivarius* reduced the biomass of both mono-species biofilms of *S. mutans* and *C. albicans* and multispecies biofilm. Rossoni et al. [69] evaluated *L. fermentum* 20.4, *L. rhamnosus* 5.2 and *L. paracasei* 28.4 strains, reducing the biofilm development of *C. albicans* ATCC 18804 and clinical isolates. James et al. [70] have evaluated a multistrain LAB combinations that exerted high effectiveness against *C. albicans* biofilms. The obtained results in the antibiofilm activity on the tested LAB strains in our study show that eight of the strains possess better antibiofilm properties against *S. mutans* than the LAB strains evaluated by other authors cited above. Also, the antibiofilm activity against *C. albicans* was noticeably high, which suggests an effective influence from the LAB strains against biofilm formation from the yeast pathogen.

The results from the co-cultivation, co-aggregation and antibiofilm activity of the studied LAB strains were compared for correlation among them using Pearson’s correlation. The obtained data indicate a positive correlation between the antagonistic activity of the studied strains against both test pathogens in co-cultivation and antibiofilm activity. A positive correlation was reported between the co-aggregation and antibiofilm activity assays of the LAB strains against *C. albicans*. No positive correlation was determined between the co-aggregation ability and the antibiofilm activity of the studied strains against *S. mutans*. Based on the established correlations, it can be supposed that the main antagonistic mechanisms of the LAB strains against *S. mutans* are related to their metabolic activity. The established antagonistic activity against *C. albicans* is more likely determined due to the direct physical exclusion of this opportunistic pathogen.

Supernatants naturally have an acidic pH due to the produced organic acids from LAB, like lactic and acetic acid. In the antioxidant capacity assay, it can be acknowledged that the higher the acidity is, the more antioxidant activity the CFS possess (Figure 5). The redox reaction between the CFSs and KMnO_4_ is quantitative, and the diameter of each discoloured zone is equivalent to the quantity of the antioxidant. Hanchi et al. [45] have assessed that the newly LAB-adapted KMnO_4_-agar antioxidant method is linear, accurate and repeatable at a time range between 30 min and 4 h and suitable for assaying LAB CFSs. As the assay gives data for the total antioxidant capacity, a more complex approach involving the ability of the tested LAB strains to reduce oxidative stress using free radicals in DPPH, ABTS, or Fenton reaction radical scavenging activity assays can give a better understanding of their antioxidant activity. The performed KMnO_4_ agar assay serves as preliminary screening for antioxidant capacity, and more detailed analyses for evaluation of the antioxidant activity will be included in subsequent studies of the LAB strains.

## 5. Conclusions

The results from this work provide information about the antagonistic properties, biofilm formation, and antioxidant capacity of previously isolated oral LAB strains against the common oral *pathogens S. mutans* and *C. albicans*. Five of the tested LAB strains stand out regarding their evaluated properties.

The tested *L. fermentum* N 2, TC 3-11 and NA 2-2 strains exhibited the most potential to antagonize the selected oral pathogens. Overall, *L. fermentum* TC 3-11 showed the most complex combination of characteristics among the performed analyses. *L. fermentum* N 2 showed the most expressed antagonistic activity against *S. mutans*, and *L. fermentum* NA 2-2 showed the highest capacity to inhibit oral pathogen biofilms. The tested *L. delbrueckii* subsp. *lactis* VG 2 and *W. confusa* NN 1 also showed promising characteristics. *L. delbrueckii* subsp. *lactis* VG 2 exhibited the highest self-biofilm formation and co-aggregation properties, and t *W. confusa* NN 1 exhibited the highest inhibition of *C. albicans*.

It can be acknowledged that *L. fermentum* N 2 and TC 3-11 possess significant probiotic characteristics [41] and most expressed antagonistic activities, and *L. fermentum* NA 2-2, *L. delbrueckii* subsp. *lactis* VG 2 and *W. confusa* NN 1 are also strains of interest for evaluation.

Our research on the antagonistic effects in co-culturing previously not studied LAB strains provides an expanded insight into their direct interactions with harmful microorganisms in the human oral microbiome. The established in vitro antagonistic activities can serve as a basis for successfully implementing the assessed LAB strains in products for oral healthcare.

## Figures and Tables

**Table 1:** Inhibition activity of the tested LAB strains in the spot assay against *S. mutans* and *C. albicans*.

Strain	Presence of Inhibition Zone	
	*S. mutans*	*C. albicans*
*L. fermentum* N 2	+	+
*L. fermentum* N 4-5	+	+
*W. confusa* AG 2-6	+	-
*L. curvatus* KG 12-1	+	-
*L. fermentum* TC 3-11	+	+
*L. delbrueckii* subsp. *sunkii* VG 1	+	-
*L. delbrueckii* subsp. *lactis* VG 2	+	+
*L. delbrueckii* subsp. *lactis* MK 13-1	+	-
*W. confusa* NN 1	+	+
*L. rhamnosus* NA 1-8	+	+
*L. fermentum* NA 2-2	+	+
*L. paracasei* AV 2-1	+	+

## Data Availability

Not applicable.
